# Laparoscopic Living Donor Nephrectomy: Learning Curve Analysis Through 1446 Cases and Outcomes from 200 Consecutive Mastery-Phase Procedures—How I Do It

**DOI:** 10.3390/jcm15041363

**Published:** 2026-02-09

**Authors:** Fahim Kanani, Moran Kozin, Yael Ben Avraham, Efrat Avitan, Michael Gurevich, Eviatar Nesher, Aviad Gravetz

**Affiliations:** 1 Department of Transplantation, Beilinson Medical Centre, Sackler Faculty of Medicine, Tel Aviv University, Tel Aviv 6997801, Israel; moran3606@gmail.com (M.K.); 93yaelba@gmail.com (Y.B.A.); efrat12daniel@gmail.com (E.A.);; 2 Department of Abdominal Organs Transplantation, Rabin Medical Center—Beilinson Campus, Gray Faculty of Medicine and Health Sciences, Tel Aviv University, Derech Ze’ev Jabotinsky 39, Petah Tikva 4941492, Israel

**Keywords:** living donor nephrectomy, laparoscopic surgery, learning curve, CUSUM analysis, kidney transplantation

## Abstract

**Background:** Laparoscopic living donor nephrectomy is a standard approach for kidney procurement, yet optimal technique and learning curve trajectories remain incompletely characterized. We present a high-volume single-center experience with standardized transperitoneal laparoscopic donor nephrectomy and CUSUM-based learning curve analysis. **Methods:** Retrospective analysis of 1446 consecutive laparoscopic living donor nephrectomies performed by six surgeons between January 2015 and December 2024. Learning curve analysis used the cumulative sum (CUSUM) methodology to identify proficiency phases. The most recent 200 consecutive cases, representing mature institutional performance, were analyzed for detailed outcomes. The surgical technique employed a transperitoneal approach with the GelPOINT^®^ Advanced Access Platform for kidney extraction via an offset Pfannenstiel incision. **Results:** CUSUM analysis identified case 669 as the inflection point, defining four phases: Phase I (initial learning, cases 1–250, *n* = 250, 154.6 ± 35.9 min), Phase II (rapid improvement, cases 251–669, *n* = 419, 136.7 ± 32.6 min), Phase III (consolidation, cases 670–1000, *n* = 331, 118.0 ± 30.1 min), and Phase IV (mastery, cases 1001–1446, *n* = 446, 101.5 ± 26.2 min). Overall operative time decreased from 154.6 to 96.8 min (37.4% reduction, *p* < 0.001). In the 200-case mastery-phase cohort, mean operative time was 96.8 ± 25.5 min with warm ischemia time of 3.8 ± 1.2 min. There were no conversions to open surgery (0%), no intraoperative complications, and one major postoperative complication (0.5%, Clavien–Dindo ≥ IIIa). Left kidney procurement was performed in 99.5% of cases. Among male donors (*n* = 86), systematic orchalgia surveillance demonstrated 46.5% prevalence at 1 month, declining to 36.0% at 1 year, and 7.0% at a 5-year follow-up. **Conclusions:** This high-volume single-center experience demonstrates favorable outcomes in laparoscopic living donor nephrectomy with CUSUM-defined proficiency phases extending beyond 1000 cases. The outcomes observed likely reflect the combined effects of institutional volume, team experience, and standardized technique. Multi-center validation is required before generalizing these results.

## 1. Introduction

Living donor kidney transplantation (LDKT) remains the optimal treatment for end-stage renal disease, offering superior long-term graft survival, reduced waiting times, and better overall recipient outcomes compared to deceased donor transplantation [1,2]. The critical limitation to expanding living donation has historically been donor morbidity associated with traditional open nephrectomy, which required large flank incisions, prolonged recovery times, and significant postoperative pain [3].

The paradigm of living donor nephrectomy was fundamentally transformed in 1995 when Ratner et al. first described laparoscopic living donor nephrectomy (LDN) at the Johns Hopkins Medical Center [4]. This minimally invasive approach demonstrated clear advantages over open surgery, including reduced postoperative pain, shorter hospital stays, faster return to normal activities, and superior cosmetic outcomes while maintaining equivalent graft function and recipient outcomes [5,6]. The widespread adoption of laparoscopic techniques has been instrumental in increasing donor willingness to participate in living donation programs worldwide [7].

Since the initial description, laparoscopic donor nephrectomy has undergone continuous technical refinements and evolution. Various approaches have been developed, including pure laparoscopic, hand-assisted laparoscopic (HALDN), retroperitoneal, and, more recently, robotic-assisted techniques [8,9]. Each approach aims to further minimize donor morbidity while ensuring optimal graft procurement. Robotic-assisted laparoscopic donor nephrectomy (RDN) has gained particular traction, offering enhanced visualization, improved ergonomics, and reduced surgeon fatigue, with reported complication rates of approximately 9% and minimal conversion to open surgery [10,11].

Despite these advances, laparoscopic donor nephrectomy remains a technically demanding procedure with a documented learning curve. Studies have demonstrated that surgical proficiency, defined by decreased operative times and reduced complication rates, is typically achieved after 30–100 cases, depending on the technique and surgeon experience [7,12]. The learning curve is particularly crucial in donor surgery, where the primary consideration must always be donor safety, as healthy individuals undergo surgery solely to benefit others.

The importance of institutional experience and standardized techniques cannot be overstated. High-volume centers performing ≥50 laparoscopic donor nephrectomies annually have demonstrated superior outcomes with reduced operative times, lower complication rates, and improved donor satisfaction [13]. Furthermore, specific technical modifications and institutional protocols have been shown to significantly impact both learning curves and long-term outcomes [14].

Our institution has been performing laparoscopic living donor nephrectomy since 2015, during which time we have developed specific technical modifications aimed at enhancing donor safety while maintaining optimal graft quality. These modifications include refinements in extraction site selection, specific approaches to ensure minimal complications, and a completely laparoscopic technique with carefully developed safety protocols. Through our experience with 1446 consecutive cases, we have achieved favorable outcomes with low complication rates, reflecting the combined effects of high institutional volume, standardized technique, and dedicated team experience.

The objectives of this study are threefold: (1) to provide a detailed description of our institutional technique for laparoscopic donor nephrectomy, including the use of the GelPOINT^®^ Advanced Access Platform and offset Pfannenstiel extraction site; (2) to analyze the institutional learning curve using cumulative sum (CUSUM) methodology across 1446 consecutive cases performed by six surgeons, identifying statistically derived proficiency phases; and (3) to evaluate safety and efficacy through comprehensive analysis of our most recent 200 consecutive mastery-phase cases.

By examining the institutional learning curve with rigorous statistical methodology, we aim to provide insights for centers seeking to establish or refine their living donor programs while maintaining the highest standards of donor safety.

## 2. Methods

### 2.1. Study Design and Objectives

This study comprises two components: (1) a learning curve analysis of 1446 consecutive laparoscopic living donor nephrectomies performed at our institution between January 2015 and December 2024, and (2) a detailed outcomes analysis of the most recent 200 consecutive cases performed between June 2023 and December 2024, representing the post-plateau “mastery phase” as defined by our learning curve analysis.

The primary objectives were as follows: (a) to characterize the institutional learning curve and identify case-volume thresholds for proficiency phases; (b) to report operative and perioperative outcomes achieved during the mastery phase; and (c) to describe our standardized surgical technique in detail.

Data were collected prospectively in our institutional transplant registry, which systematically records demographic, operative, and follow-up variables for all living donors. This study was approved by the Medical Center Institutional Review Board (**-0804-23, approved 5 December 2023; extended 21 January 2025). As a retrospective analysis of registry data, the requirement for individual informed consent was waived by the IRB. The study was conducted in accordance with the Declaration of Helsinki.

All potential living kidney donors underwent comprehensive evaluation according to international transplant guidelines. The evaluation protocol included medical, psychological, and surgical assessments performed by senior transplant surgeons with at least 5 years of experience in kidney transplantation. Inclusion criteria comprised all adult donors (≥18 years) who were medically cleared for elective nephrectomy, including both living-related and altruistic donors. We applied no additional exclusion criteria beyond standard donor evaluation protocols.

We collected comprehensive baseline demographic data, including age, sex, body mass index (BMI), donor–recipient relationship (categorized as genetically related, emotionally related/spouse, or altruistic), medical comorbidities, previous abdominal surgery, and preoperative renal function (serum creatinine and estimated glomerular filtration rate calculated using the CKD-EPI equation). Our institutional protocol strongly favored left kidney procurement based on the longer renal vein length, with right nephrectomy performed only when the left kidney was contraindicated.

Operative data collection included detailed vascular anatomy (number of renal arteries and veins), total operative time (skin incision to closure), warm ischemia time (from arterial clamping to cold perfusion), estimated blood loss, intraoperative complications, and any conversions to open surgery. All procedures utilized our standardized laparoscopic technique with the GelPOINT^®^ Advanced Access Platform for kidney extraction.

We tracked postoperative outcomes through a standardized follow-up protocol. Donors were evaluated at 1 week, 1 month, 3 months, 6 months, 1 year, and annually thereafter. We documented the length of hospital stay, postoperative complications classified by the Clavien–Dindo system, and readmission rates at 30 and 90 days. Return to work was assessed through patient self-report at follow-up visits. Long-term complications, particularly incisional hernias and chronic pain, were evaluated with a minimum 3-year follow-up period. For male donors, we systematically assessed testicular pain (orchalgia) at each follow-up visit using a standardized questionnaire.

### 2.2. Statistical Analysis

Continuous variables are presented as mean ± standard deviation and median (interquartile range); categorical variables are presented as frequencies and percentages.

Learning curve analysis was performed using the cumulative sum (CUSUM) methodology. CUSUM values were calculated as the running total of deviations from the overall mean operative time, plotted against consecutive case numbers. CUSUM analysis was performed on the entire institutional series as an aggregate, treating consecutive cases chronologically regardless of operating surgeon. Individual surgeon learning curves were not analyzed separately; the curve therefore represents institutional rather than individual proficiency development. The CUSUM inflection point—where the curve transitions from rising (performance below target) to falling (performance exceeding target)—was identified as the case number at the maximum CUSUM value. This inflection point, combined with analysis of 50-case moving averages, was used to define discrete proficiency phases. A priori, we defined the “mastery phase” as the period following CUSUM plateau stabilization.

Comparisons between phases were performed using independent samples *t*-tests. Statistical significance was set at *p* < 0.05. The most recent 200 consecutive cases, representing mature institutional performance, were analyzed separately for detailed outcome reporting. All analyses were conducted using Python 3.11 (scipy, numpy, and pandas libraries) and SPSS version 28.0 (IBM Corp., Armonk, NY, USA).

Surgical Technique: Laparoscopic Left Living Donor Nephrectomy—Institutional Protocol

Surgical Technique

**Figure 1:**
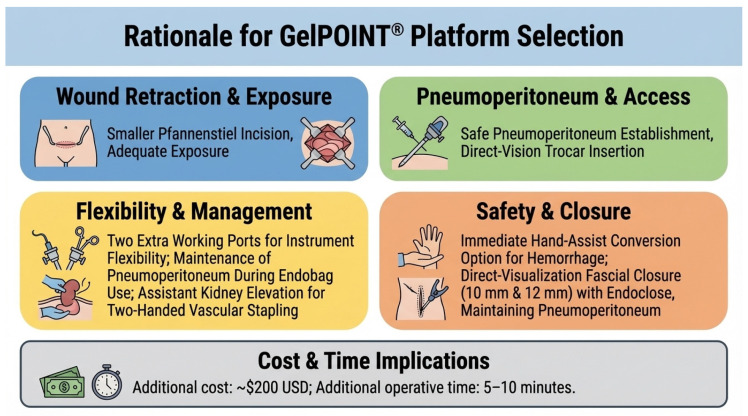
GelPOINT^®^ use rationale. See Figure S1 in the Supplementary Materials.

All procedures followed a standardized 12-step protocol developed and refined over the study period. The complete technical description, including specific instrumentation, anatomical landmarks, and safety checkpoints, is provided in the Surgical Technique Section. Key procedural steps are illustrated in Figure 1.

Overview of Our Approach

**Figure 2:**
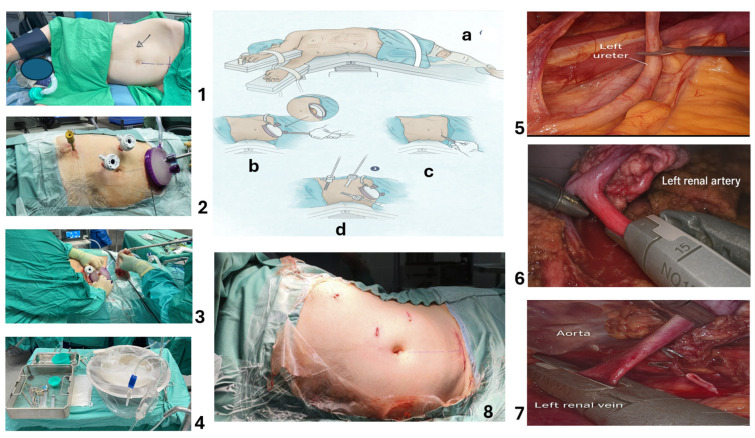
GelPOINT^®^ Advanced Access Platform for laparoscopic living donor nephrectomy—a refined technique. (1). Patient positioning—modified left lateral decubitus position (“modified jackknife”) to maximize the working space between the costal margin and iliac crest. The left shoulder rests comfortably in the groove of the operating table, and the left arm is supported on a padded shelf. All incision sites are marked in the supine position before lateral positioning to ensure symmetry: the suprapubic incision is marked 1 cm cranial to the pubic symphysis and 6–7 cm laterally on each side. (2). GelPOINT^®^ placement and port setup—after an under-vision opening of the anterior rectus fascia and gentle muscle separation, the GelPOINT^®^ Advanced Access Platform is inserted through a low suprapubic Pfannenstiel incision. The peritoneum and preperitoneal fat are opened under direct vision, carefully avoiding bladder injury. Three additional trocars are placed: 11 mm (epigastric camera port), 12 mm (left lower quadrant working port), and 5 mm (left subcostal retraction port). (3). Kidney extraction—once hilar transection is complete, the kidney is extracted immediately through the GelPOINT^®^ platform, preventing traction injury and minimizing ischemia time. The port system is designed to avoid kidney compression and facilitate a smooth, atraumatic graft removal. (4). Graft preservation setup—the Celsior^®^ preservation solution (or an equivalent preservation fluid) is prepared in a sterile basin with ice. The kidney is flushed immediately after retrieval to optimize graft viability before transfer to the recipient operating room. Middle panel—surgical illustration summarizing key steps of positioning, GelPOINT^®^ insertion, and trocar placement; a: left lateral decubitus with modified jack-knife; b: low Pfannenstiel incision; c: GelPOINT^®^ placement after fascial entry; and d: trocar positions with subcostal, lateral, and suprapubic access. (5). Ureter division—the left ureter is divided just above the iliac artery crossing, preserving periureteric vascular supply to ensure graft viability. (6). Inflow control—left renal artery stapling—the left renal artery is carefully dissected circumferentially and divided using a laparoscopic vascular stapler, maximizing arterial length for optimal transplantation. (7). Outflow control—left renal vein stapling—the left renal vein is stapled flush with its aortic confluence to obtain maximum venous length while maintaining donor safety. (8). Postoperative incisions.

Our institutional technique for laparoscopic living donor nephrectomy represents a refined, completely laparoscopic approach developed over 1446 consecutive cases since 2015. We have developed specific technical modifications that prioritize donor safety while ensuring optimal graft procurement. Our approach utilizes a standardized transperitoneal technique with a unique extraction site configuration and specialized safety protocols that have resulted in minimal complications and no serious adverse events (Figure 2).

Preoperative Considerations and Patient Selection

Inclusion Criteria

All potential donors undergo comprehensive medical, psychological, and surgical evaluation according to established guidelines. Our specific inclusion criteria include:

- Body Mass Index (BMI): We accept donors with BMI up to 30 kg/m^2^, with careful consideration of body habitus and distribution of adipose tissue.
- Previous Abdominal Surgery: Not an absolute contraindication; we provide detailed informed consent regarding potential increased complexity and conversion risk.
- Anatomical Considerations: We strongly prefer left kidney procurement due to the longer renal vein (typically 6–8 cm vs. 2–3 cm on the right).

Kidney Selection Philosophy

Our institutional protocol strongly favors left kidney procurement. The rationale is threefold: (1) the longer left renal vein (typically 6–8 cm vs. 2–3 cm on the right) facilitates safer donor hilar dissection and recipient venous anastomosis; (2) the shorter right renal vein increases donor bleeding risk and recipient venous thrombosis risk; and (3) standardization of left-sided procurement optimizes team efficiency and reduces procedural variability.

Our preference hierarchy is:

- Left kidney with normal anatomy;
- Left kidney with multiple arteries (up to 3 vessels);
- Right kidney only when the left kidney demonstrates significantly inferior function or anatomy.

Notably, for related donors with inter-kidney asymmetry, right-sided procurement is considered but remains rare in our experience. We do not perform routine baseline scintigraphy; kidney selection is based on computed tomography angiography and estimated glomerular filtration rate, with the assumption that symmetric anatomy implies symmetric function.

We acknowledge that this approach represents an institutional preference. While the longer left renal vein offers technical advantages, high-volume centers have reported equivalent outcomes with right-sided procurement when anatomically indicated.

Anesthesia and Initial Setup

Anesthetic Management

- General Anesthesia: Standardized protocol with endotracheal intubation.
- Muscle Relaxation: Complete neuromuscular blockade maintained throughout the procedure.
- Ventilation: Pressure-controlled ventilation with PEEP 5–8 cmH_2_O to optimize visualization.
- CO_2_ Monitoring: End-tidal CO_2_ monitoring with adjustments for pneumoperitoneum effects.

Urinary Catheterization

- Foley Catheter: Silicone catheter of 16–18 Fr with 10 mL balloon.
- Bladder Decompression: Critical for safe suprapubic incision placement.
- Sterile Drainage: Closed drainage system maintained throughout procedure.

Patient Positioning and Surgical Preparation

Skin Marking and Positioning Strategy

Supine Phase Preparation—before positioning, with the patient supine, we perform critical skin marking:

- Extraction Site Marking: Transverse suprapubic incision site marked 1 cm cephalad to pubic symphysis (for optimal cosmetic result, ensuring incision remains below underwear line).
- Port Placement Marking: All trocar sites marked based on patient anatomy.
- Anatomical Landmarks: Identification of anterior superior iliac spines, umbilicus, and costal margins.

Lateral Decubitus Positioning

Patient Positioning Protocol:

- Position: Modified right lateral decubitus (left side up).
- Table Flexion: Moderate table flexion to open the space between the costal margin and the iliac crest.
- Support Systems:

○ Axillary roll placement to protect the brachial plexus.
○ Kidney rest positioned at the level of the 12th rib.
○ Gel padding for all pressure points.

- Arm Positioning: The left arm is placed in extension and supported on a padded arm board above the patient’s torso to maintain stability and prevent nerve compression. The right arm is tucked at the patient’s side, with the shoulder carefully protected to avoid traction injury.
- Leg Positioning: Pillow between legs, lower leg flexed, and upper leg straight.
- Security: Patient secured with wide tape across the hip and shoulder.

Surgical Access and Trocar Placement

Extraction Site Creation and GelPOINT^®^ (Figure 1 and Figure 2) Setup

Suprapubic Pfannenstiel Incision: Our extraction site represents a key technical modification that enhances both safety and cosmetic outcomes:

Skin Incision:

- Location: Transverse suprapubic incision, 6–8 cm in length.
- Position: One centimeter cephalad to the pubic symphysis for cosmetic optimization.
- Technique: Sharp dissection through skin and subcutaneous tissue using electrocautery.

Fascial Approach:

- Critical Modification: While the skin incision is placed low for cosmetic reasons, the fascial incision is intentionally placed 2–3 cm higher (more cephalad).
- Rationale: This offset technique minimizes the risk of bladder injury while maintaining the cosmetic advantage.
- Dissection: Sharp dissection through the anterior rectus fascia.
- Muscle Layer Management:
- Rectus Muscle Mobilization: Both rectus muscles are gently mobilized laterally from the fascia using blunt dissection.
- Preperitoneal Space: Access gained through the natural fibrous tissue plane between the rectus muscles.
- Peritoneal Entry: Peritoneum identified and opened under direct visualization using sharp dissection.

GelPOINT^®^ Advanced Access Platform Setup:

- Device: Applied Medical GelPOINT^®^ Advanced Access Platform (10 cm in diameter).
- Insertion: Platform inserted through the Pfannenstiel incision.
- Seal Verification: Ensuring a circumferential seal around wound edges.
- Assistant Ports: Two 12 mm assistant ports placed within the GelSeal^®^ cap (Applied Medical, Rancho Santa Margarita, CA, USA).
- Insufflation: Primary insufflation port connected to maintain pneumoperitoneum at 15 mmHg.

Laparoscopic Port Placement

Primary Camera Port (11 mm):

- Location: One to two centimeters above the umbilicus at the epigastrium.
- Trocar Type: Eleven-millimeter optical trocar (Ethicon ENDOPATH^®^ XCEL^®^).
- Insertion: Under direct visualization through GelPOINT^®^ after initial insufflation.
- Camera: High-definition 10 mm laparoscope (Olympus^®^ or Karl Storz^®^ 3D system).

Working Port Configuration:

- Left Lower Quadrant Port (12 mm):

○ Location: Halfway between the anterior superior iliac spine and the umbilicus.
○ Purpose: Primary working port for dissection instruments.
○ Trocar: Twelve-millimeter bladeless trocar (Ethicon ENDOPATH^®^).

- Left Upper Quadrant Port (5 mm):

○ Location: Left subcostal, medial to midclavicular line.
○ Purpose: Retraction and secondary dissection.
○ Trocar: Five-millimeter Step^®^ trocar (Covidien).

Visualization System

Optical Equipment:

- Camera System: Olympus VISERA ELITE III^®^ or Karl Storz IMAGE1 S™ 3D system (Karl Storz, Tuttlingen, Germany).
- Resolution: 4K Ultra HD with 3D visualization capability.
- Lighting: Xenon or LED light source with automatic light control.
- Monitor Configuration: Dual 32-inch 4K monitors positioned for optimal surgeon ergonomics.

Instrumental Arsenal and Energy Devices

Primary Dissection Instruments

Harmonic Scalpel Technology:

- Device: Ethicon Harmonic ACE^®^ (Ethicon, Cincinnati, OH, USA)+ Shears or Harmonic SYNERGY^®^ Hook
- Technology: Ultrasonic energy at 55,500 Hz frequency.
- Advantages:

○ Simultaneous cutting and coagulation.
○ Minimal lateral thermal spread (0.5–2 mm).
○ Reduced smoke production.
○ Precise tissue dissection with minimal char formation.

- Settings: Power level 3–5 depending on tissue type and thickness.
- Clipping Systems:
- Primary: Ethicon LIGACLIP^®^ EXTRA Large (5 mm clips).
- Secondary: Aesculap CHALLENGER^®^ medium-large clips.
- Application: Secure vascular and ductal structure control.
- Grasping and Retraction:
- Atraumatic Graspers: Ethicon HARMONIC^®^ curved scissors.
- Bowel Graspers: Karl Storz CLICKLINE^®^ atraumatic graspers.
- Needle Drivers: Ethicon MEGADYNE^®^ laparoscopic needle drivers for suturing.

Detailed Step-by-Step Surgical Technique

Phase 1: Initial Exploration and Colon Mobilization

Systematic Inspection—Upon achieving pneumoperitoneum and completing port placement, we begin with systematic inspection:

- General Survey: Complete visualization of the peritoneal cavity, identifying any adhesions from previous surgery.
- Anatomical Orientation: Identification of key landmarks, including the splenic flexure, the descending colon, and the left paracolic gutter.
- Pathology Assessment: Ruling out unexpected pathology that might contraindicate donation.

Freno-Omento-Colic Ligament Division:

- Identification: The freno-omento-colic ligament connecting the splenic flexure to the diaphragm.
- Division Technique: Using a harmonic scalpel at a power setting of 3–4.
- Safety Consideration: Careful attention to avoid splenic capsular injury.

Left Colon Mobilization Along Toldt’s Line—This represents a critical step that sets the foundation for safe retroperitoneal access:

- Anatomical Landmarks:

○ Medial border: Toldt’s white line (embryological fusion plane).
○ Lateral border: Paracolic gutter.
○ Superior limit: Splenic flexure.
○ Inferior limit: Sigmoid colon.

- Dissection Technique:

○ Initial Incision: Sharp division of the peritoneum along Toldt’s line using the harmonic scalpel.
○ Plane Development: Blunt and sharp dissection, maintaining the correct areolar tissue plane.
○ Critical Safety Point: Avoiding penetration into retroperitoneal fat, which can lead to bleeding and loss of anatomical planes.
○ Mesocolon Preservation: Maintaining mesocolic vessels intact to ensure colon viability.

Key Technical Points for Colon Mobilization:

- Pressure Dynamics: Maintaining pneumoperitoneum at 15 mmHg to optimize exposure.
- Retraction Strategy: Gentle medial retraction of the colon without excessive tension.
- Hemostasis: Immediate control of any bleeding points with clips or a harmonic scalpel.
- Anatomical Respect: Avoiding kidney mobilization at this stage to prevent medial displacement.

Phase 2: Retroperitoneal Access and Upper Pole Dissection

Gerota’s Fascia Approach:

- Identification: Recognition of the glistening Gerota’s fascia overlying the kidney.
- Initial Entry: Careful incision of Gerota’s fascia at the upper pole.
- Plane Development: Establishing the correct plane between Gerota’s fascia and perirenal fat.

Perirenal Fat Pad Dissection:

- Technique: Systematic dissection of perirenal fat using a combination of blunt and sharp dissection.
- Anatomical Goal: Exposure of renal capsule without capsular injury.
- Superior Extent: Dissection carried to the left subphrenic recess, where intraperitoneal fluid may be encountered.

Splenorenal Ligament Division:

- Anatomical Structure: Fibrous ligament connecting the spleen to the kidney region.
- Division Technique: Sharp division with a harmonic scalpel.
- Safety Measures: Careful attention to avoid splenic injury or excessive bleeding.

Phase 3: Adrenal Gland and Vascular Identification

Left Adrenal Gland Exposure:

- Anatomical Recognition: Golden-yellow glandular tissue superior and medial to the upper kidney pole.
- Dissection Plane: Maintaining a plane between the adrenal gland and the kidney.
- Safety Protocol: Avoiding adrenal gland injury, which can result in significant bleeding.

Adrenal Vein Identification and Dissection:

- Anatomical Course: Short vein (typically 1–2 cm) draining directly into the renal vein.
- Dissection Technique: Careful dissection using a fine dissector (Maryland, harmonic scalpel).
- Preparation for Division: Ensuring adequate length for safe clipping.

Phase 4: Hilar Vascular Dissection

Left Renal Vein Exposure—The renal vein exposure represents one of the most critical phases:

- Initial Identification: Recognition of the renal vein coursing medially toward the IVC.
- Systematic Dissection:

○ Superior border cleared first.
○ Inferior border dissection with attention to lumbar veins.
○ Circumferential clearing to allow stapler passage.

Gonadal Vein Management:

- Anatomical Course: Tracing the gonadal vein from its drainage point into the renal vein.
- Proximal Dissection: Skeletonization near renal vein junction.
- Division Strategy: Initial division near the renal vein, secondary division at the pelvic brim.

Critical “Danger Triangle/Golden Triangle (Triangolo Dóro)” Dissection—This area is bounded by [15,16]:

- Superior: Lower pole of the kidney.
- Medial: Renal vein.
- Lateral: Large vessels (aorta and IVC).

**Figure 3:**
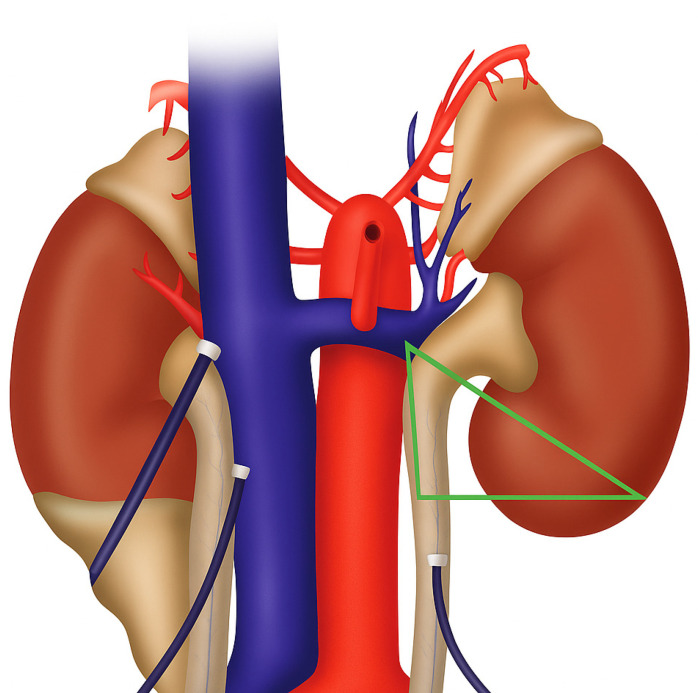
The “Golden Triangle” (Triangolo d’Oro): critical dissection zone in laparoscopic donor nephrectomy. The green triangle delineates the anatomical boundaries of the high-risk dissection area, bounded superiorly by the renal vein, laterally by the ureter (elevated on retractor), and medially by the psoas muscle. Lumbar veins traversing this zone represent the primary source of intraoperative hemorrhage. Meticulous, sharp dissection with minimal energy application and systematic clip ligation of lumbar tributaries is essential for safe hilar exposure. See Supplementary Figure S2 for a detailed illustration of the dissection technique and lumbar vein management.

Technical Approach to Danger Triangle: Delicate, sharp dissection is needed for safe identification and ligation/division of lumbar veins (Figure 3) bordered by the ureter (lifted), renal vein, and the psoas muscle.

- Dissection Philosophy: Extremely delicate approach with minimal tissue handling.
- Lumbar Vein Awareness: Recognition that small lumbar veins may traverse this area.
- Bleeding Prevention: Immediate recognition and control of any venous bleeding.
- Instrument Selection: Use of the finest dissection instruments with minimal energy application.

Phase 5: Ureteral Mobilization

Ureteral Identification:

- Anatomical Location: Midway between the lower kidney pole and the common iliac vessel crossing.
- Recognition: Peristaltic activity and characteristic white, cord-like appearance.

Ureteral Dissection Technique—Our technique emphasizes preservation of the ureteral blood supply:

- Periureteral Tissue Preservation: Maintaining the adventitial layer with its blood supply.
- “Meso-ureter” Concept: Preserving the delicate vascular network surrounding the ureter.
- Thermal Injury Prevention:

○ No direct energy application to the ureter.
○ Minimum 5 mm of clearance when using the harmonic scalpel.
○ Sharp dissection when in close proximity.

Dissection Extent:

- Proximal: To the renal pelvis.
- Distal: To the common iliac vessel crossing.
- Safety Zone: Avoiding dissection beyond the common iliac vessels to prevent devascularization.

Phase 6: Renal Artery Dissection

Renal Artery Exposure:

- Anatomical Approach: Posterior and slightly inferior to the renal vein.
- Lymphatic Tissue Management: Careful dissection of abundant lymphatic tissue around the aorta.
- Lymph Node Handling: Selective lymph node dissection to expose the arterial origin.

Arterial Mobilization:

- Circumferential Clearing: Dissection of 360 degrees to allow stapler passage.
- Origin Exposure: Clear visualization of the aortic origin.
- Length Optimization: Maximizing arterial length for transplantation.

Inter-Hilar Space Creation:

- Critical Separation: Establishing clear space between artery and vein.
- Stapler Accommodation: Ensuring adequate space for safe stapler passage.
- Safety Verification: Confirming no posterior structures in the stapler path.

Phase 7: Final Mobilization and Preparation

Distal Ureteral and Gonadal Vein Division:

- Gonadal Vein: Secondary division at pelvic brim between clips.
- Ureteral Division: Above clip placement, ensuring adequate length.

Retroperitoneal Fat Mobilization:

- Medial Mobilization: Freeing the kidney from retroperitoneal attachments.
- Posterior Dissection: Careful attention to avoid lumbar vessels.
- Lateral Release: Complete mobilization for extraction.

Pre-Extraction Setup:

- Endo-Bag Preparation: Introduction of the extraction bag through GelPOINT^®^.
- Kidney Positioning: Careful placement ensuring no vascular kinking.
- Final Inspection: Verification of complete mobilization.

Phase 8: Vascular Division and Extraction

Vascular Stapling Protocol—Our institution uses the Ethicon ECHELON FLEX™ (Ethicon, Cincinnati, OH, USA) Powered Vascular Stapler system:

Device Specifications:

- Stapler: Ethicon ECHELON FLEX™-powered vascular stapler.
- Cartridge: Gold cartridge (3.5 mm staple height) for vascular tissue.
- Firing: Powered articulation with consistent compression and firing.

Division Sequence:

1. Renal Artery First:

○ Rationale: Immediate cessation of perfusion.
○ Technique: Single firing with adequate proximal margin.
○ Verification: Complete division with hemostatic staple line.

2. Renal Vein Second:

○ Division Level: Just proximal to the adrenal vein stump.
○ Rationale: Maximizing vein length for transplantation.
○ Lymphatic Consideration: A large lymph node frequently present between the adrenal gland and the renal vein may be included in the staple line.

Extraction Protocol:

- Bag Closure: Gradual closure of the extraction bag while maintaining the kidney position.
- GelPOINT^®^ Extraction: Atraumatic removal through suprapubic incision.
- Immediate Assessment: Rapid evaluation of graft quality and vascular integrity.

Hemostasis and Closure Protocol

Systematic Hemostasis

Inspection Protocol:

- Pneumoperitoneum Reduction: Reducing pressure to 5–8 mmHg to identify venous bleeding.
- Systematic Review:

○ Renal fossa inspection;
○ Vascular stump evaluation;
○ Colon and spleen assessment;
○ Port site evaluation.

Hemostatic Agents:

- Primary: Electrocautery for pinpoint bleeding.
- Secondary: Hemostatic clips for specific vessels.
- Adjunctive: Topical hemostatic agents (Surgicel^®^ Ethicon, Cincinnati, OH, USA or Gelfoam^®^ (Pfizer, Kalamazoo, MI, USA) if needed.

Special Attention to Lymphatic Leakage

Prevention Strategy:

- Minimal Lymph Node Dissection: Only as necessary for vascular exposure.
- Clip Application: Liberal use of clips on lymphatic channels.
- Recognition: Early identification of lymphatic fluid (clear, colorless).

Management of Lymphatic Leaks:

- Small Leaks: Clip application or light electrocautery.
- Larger Leaks: Suture ligation with non-absorbable suture.
- Drainage Consideration: Selective drain placement if a significant leak is suspected.

Closure Technique

Fascial Closure:

- Suture Material: 0-Vicryl on a large needle (CT-1 or CT-2).
- Technique: Running suture with adequate tissue bites.
- Verification: Ensuring no fascial gaps.

Port Site Closure:

- 12 mm Ports: Fascial closure mandatory to prevent hernia.
- 5 mm Ports: Fascial closure if extended or if fascial defect apparent.
- Suture Material: 2-0 Vicryl with Carter–Thomason closure device.

Skin Closure:

- Technique: 4-0 Monocryl subcuticular suture.
- Adhesive: Dermabond^®^ (Ethicon, Somerville, NJ, USA) skin adhesive for additional security.
- Dressing: Sterile transparent dressing.

Postoperative Considerations

Immediate Recovery Protocol

- Pain Management: Multimodal analgesia protocol.
- Early Mobilization: Ambulation within 6–8 h.
- Diet Advancement: Clear liquids advancing to regular diet as tolerated.
- Discharge Planning: Typically postoperative day 1–2.

Follow-up Protocol

- Short-term: 1 week, 1 month, and 3 months.
- Long-term: Annual follow-up with renal function assessment.
- Complication Monitoring: Systematic tracking of all complications.

Technical Innovations and Safety Measures

Unique Aspects of Our Technique

1. Offset Incision Strategy: Skin incision placed lower than fascial incision for optimal cosmetics without compromising safety.
2. GelPOINT^®^ Utilization: Single extraction site serving dual purpose as extraction site and primary access port.
3. Systematic Hemostasis Protocol: Standardized approach to identifying and managing bleeding.
4. Lymphatic Preservation: Minimal lymph node dissection to reduce chylous complications.
5. Thermal Injury Prevention: Strict protocols for energy device usage near the ureter.

Quality Metrics and Safety Benchmarks

Operative Time Targets:

- Beginner: <180 min;
- Proficient: <150 min;
- Master: <120 min.

Safety Metrics:

- Conversion Rate: <2%;
- Major Complication Rate: <5%;
- Ureteral Complication Rate: <1%;
- Readmission Rate: <3%.

This detailed technique description represents our institutional standard for laparoscopic living donor nephrectomy, refined through 1446 consecutive cases with continuous quality improvement and safety optimization.

## 3. Results

### 3.1. Patient Demographics and Baseline Characteristics

A total of 200 consecutive laparoscopic living donor nephrectomies were performed during the study period. Donor demographics and baseline characteristics are summarized in Table 1. The mean age was 41.6 ± 8.6 years (range 25–66 years) with a median of 42 years (IQR 35–48). The majority of donors were female (114 patients, 57.0%) compared to male donors (86 patients, 43.0%).

The mean BMI was 24.8 ± 3.0 kg/m^2^ (range 16.2–32.9 kg/m^2^). BMI distribution showed 102 patients (53.4%) with BMI < 25 kg/m^2^, 83 patients (43.5%) with BMI 25–30 kg/m^2^, and only 6 patients (3.1%) with BMI > 30 kg/m^2^. The donor population demonstrated minimal comorbidities, with hypertension present in 1 patient (0.5%), smoking history in 12 patients (6.3%), asthma in 6 patients (3.1%), and thyroid disease in 4 patients (2.1%). No donors had diabetes mellitus. Previous abdominal surgery was documented in 47 patients (24.6%).

Regarding the relationship to the recipient, 145 donors (76.0%) were related donors, 35 (18.3%) were spouses, and 11 (5.7%) were altruistic donors. Preoperative renal function demonstrated excellent baseline kidney function with a mean estimated glomerular filtration rate of 95.2 ± 15.8 mL/min/1.73 m^2^ and a mean serum creatinine of 0.85 ± 0.18 mg/dL (Table 1).

### 3.2. Operative Characteristics and Outcomes

Operative characteristics and technical outcomes are detailed in Table 2. Left kidney procurement was performed in 199 cases (99.5%), with only one right kidney procurement (0.5%), consistent with our institutional protocol. Renal vascular anatomy showed a single renal artery in 164 cases (86.3%) and multiple renal arteries in 26 cases (13.7%), including 22 cases (11.6%) with two arteries and 4 cases (2.1%) with three arteries. Multiple renal veins were present in three cases (1.6%).

Mean operative time was 96.8 ± 25.5 min with a median of 93 minutes (IQR 85–120 min) and a range of 65–185 min. Mean warm ischemia time was 3.8 ± 1.2 min with a median of 4 min (IQR 3–4 min). Mean estimated blood loss was 85.3 ± 45.2 mL with a median of 75 mL (IQR 50–100 mL). Mean length of hospital stay was 3.5 ± 1.8 days with a median of 3 days (IQR 2–4 days).

No intraoperative complications occurred in this series. Specifically, there were no conversions to open surgery (0%), no vascular injuries (0%), no bowel injuries (0%), no splenic injuries (0%), and no intraoperative bleeding requiring intervention (0%). All 200 procedures utilized the Pfannenstiel suprapubic extraction site (100%) and complete laparoscopic approach (100%) with no hand-assisted procedures (Table 2).

### 3.3. Postoperative Complications and Outcomes

Postoperative complications and outcomes are presented in Table 3. Overall, complications occurred in 12 patients (6.0%). According to the Clavien–Dindo classification, Grade I complications occurred in eight patients (4.0%), Grade II complications in three patients (1.5%), and Grade III complications in one patient (0.5%). No Grade IV or Grade V complications occurred (0%).

Specific wound-related complications included superficial surgical site infection in two patients (1.0%) and seroma formation in one patient (0.5%). No deep surgical site infections occurred (0%). With a minimum 3-year follow-up, no trocar site hernias (0%) or Pfannenstiel incision hernias (0%) were identified.

Intra-abdominal complications were minimal, with one patient (0.5%) developing an intra-abdominal collection. No patients experienced bleeding requiring intervention (0%) or bowel obstruction (0%). Urological complications included urinary retention in three patients (1.5%) and urinary tract infection in four patients (2.0%). No pulmonary or thromboembolic complications occurred (0%).

Among male donors (*n* = 86), testicular pain (orchalgia) was systematically assessed. At 1 month postoperatively, 40 patients (46.5%) reported orchalgia. This decreased to 31 patients (36.0%) at 1 year and further reduced to 6 ± 3 patients (7.0 ± 3.5%) at a 5-year follow-up. Chronic pain persisting beyond 3 months occurred in three patients (3.5%).

Readmission rates were low, with two patients (1.0%) requiring readmission within 30 days and three patients (1.5%) within 90 days. Mean return to work time was 18.5 ± 8.2 days with a median of 16 days (IQR 12–24 days) (Table 3).

### 3.4. Comparison with the Contemporary Literature

Table 4 presents contemporary benchmarks from high-volume laparoscopic and robotic donor nephrectomy series. Our mastery-phase operative time was 96.8 ± 25.5 min compared to 180–220 min in published series. The conversion rate was 0% (the literature range: 1.0–2.3%). The major complication rate was 0.5% (the literature range: 3.8–9.1%). Length of hospital stay was 3.5 ± 1.8 days (the literature range: 3.5–4.8 days).

### 3.5. Learning Curve Analysis

**Figure 4:**
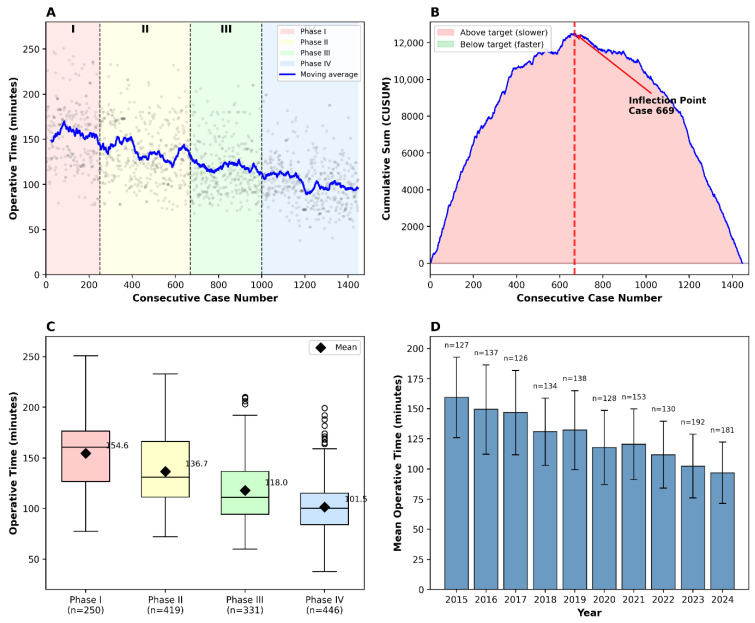
Learning curve analysis using CUSUM methodology. (**A**) Operative time by consecutive case number with 50-case moving average (blue line). Shaded regions indicate CUSUM-derived phases: Phase I (initial learning, cases 1–250, red), Phase II (rapid improvement, cases 251–669, yellow), Phase III (consolidation, cases 670–1000, green), and Phase IV (mastery, cases 1001–1446, blue). Vertical dashed lines indicate phase boundaries. (**B**) CUSUM plot demonstrating an inflection point at case 669, where cumulative performance transitions from below-target (red shading, indicating operative times above the series mean) to above-target (green shading, indicating operative times below the series mean) operative time. (**C**) Box plots showing operative time distribution by phase. Horizontal lines indicate median; boxes indicate interquartile range; whiskers indicate range; diamonds indicate mean values with numerical labels. (**D**) Annual operative time trends showing mean ± standard deviation with case volumes (*n*) for each year. CUSUM = cumulative sum.

CUSUM analysis of 1446 consecutive cases identified case 669 as the inflection point, where institutional performance transitioned from below-target to consistently above-target operative times (Figure 4B). Based on the CUSUM trajectory and moving average analysis, four distinct phases were identified (Table 5, Figure 4A–D).

Phase I (initial learning, cases 1–250, *n* = 250) demonstrated a mean operative time of 154.6 ± 35.9 min. Phase II (rapid improvement, cases 251–669, *n* = 419) showed a significant reduction to 136.7 ± 32.6 min (11.6% reduction, *p* < 0.001). Phase III (consolidation, cases 670–1000, *n* = 331) achieved a value of 118.0 ± 30.1 min (13.6% reduction, *p* < 0.001). Phase IV (mastery, cases 1001–1446, *n* = 446) reached 101.5 ± 26.2 min (14.0% reduction, *p* < 0.001).

Overall, operative time decreased from 154.6 ± 35.9 min in the first 250 cases to 96.8 ± 25.5 min in the last 200 cases, representing a 37.4% reduction (*p* < 0.001). Six surgeons contributed to both the overall cohort and the 200-case mastery-phase analysis.

### 3.6. Recipient Outcomes

First-year recipient outcomes for the 200-case mastery-phase cohort are summarized in Table S1. Delayed graft function (DGF), defined as dialysis requirement within the first post-transplant week, occurred in two recipients (1.0%). Neither case was attributable to procurement-related factors. The first involved a young recipient with long-standing type 2 diabetes mellitus, prior below-knee amputation, and severely calcified iliac arteries; following multidisciplinary team deliberation, transplantation proceeded, given intractable dialysis-related morbidity. Post-anastomosis Doppler demonstrated low diastolic flow with acute tubular necrosis (ATN) resolving after one week. The second case involved an anatomically long renal artery; surveillance Doppler at 3 h post-transplant revealed pathologic flow patterns requiring re-exploration and arterial anastomosis revision, with subsequent ATN and complete recovery.

Immediate graft function was achieved in 198 recipients (99.0%). Primary non-function did not occur. At one year, graft survival was 98.5% (197/200), with a mean serum creatinine of 1.28 ± 0.34 mg/dL and an estimated GFR of 58.4 ± 14.2 mL/min/1.73 m^2^. Ureteral complications (leak or stricture) occurred in two recipients (1.0%). One recipient (0.5%) required vascular re-intervention as described above.

## 4. Discussion

This retrospective analysis of 1446 consecutive laparoscopic living donor nephrectomies represents one of the largest single-center experiences reported, with our recent 200 cases achieving a mean operative time of 96.8 ± 25.5 min—among the most efficient reported in the contemporary literature. Our mastery-phase operative time (Median 93.6 min (IQR 85–120)) compares favorably with contemporary series reporting 180–220 min. However, these comparisons are presented for contextual benchmarking only. Direct statistical comparison is not appropriate due to heterogeneity in patient selection, surgical techniques, outcome definitions, and follow-up protocols across studies. The favorable outcomes observed in our series likely reflect the combined effects of high institutional volume, standardized technique, and case selection rather than any single technical factor. Our zero conversion rate and 0.5% major complication rate compare exceptionally favorably with published series, where conversion rates typically range from 1.08 to 2.3% [2,3] and major complications from 3.9 to 9.05% [4,5,6,7,8].

The mechanisms potentially underlying these outcomes reflect both technical innovation and institutional learning. The GelPOINT^®^ Advanced Access Platform provides several practical advantages in our technique. First, it functions as a wound retractor, enabling a smaller Pfannenstiel incision while maintaining adequate exposure. Second, it establishes pneumoperitoneum and allows subsequent trocar placement under direct vision, avoiding blind abdominal entry. Third, it provides two additional working ports through the gel cap. Fourth, pneumoperitoneum is maintained during endobag insertion and manipulation; once the kidney is placed inside the endobag, the assistant can elevate the graft, freeing both surgeons’ hands for vascular stapling—a critical safety advantage during hilar transection. Fifth, it provides an immediate safety option for hand-assisted conversion if hemorrhage control is required, without the need for additional incisions. Sixth, it permits fascial closure of the 10 mm and 12 mm port sites under direct visualization without losing pneumoperitoneum, using an endoclose device. These advantages come at a modest additional cost (~$200 USD) and add approximately 5–10 min to operative time. While we cannot determine from this non-comparative study whether outcomes would differ with alternative extraction methods, we believe these practical benefits contribute to the efficiency and safety profile observed in our series. This likely explains our ability to manage complex vascular anatomy (13.7% multiple arteries) without conversions. Combined with our offset incision technique—placing fascial entry 2–3 cm higher than the cosmetic skin incision—and strict protocols for minimal lymph node dissection and thermal injury prevention, these modifications may create a reproducible system for achieving consistent excellence [7,11,12].

**Learning Curve Interpretation**

Our CUSUM-based learning curve analysis provides a statistically rigorous characterization of proficiency development in laparoscopic donor nephrectomy. The identification of case 669 as the CUSUM inflection point—where cumulative performance transitions from below-target to consistently above-target operative time—offers an objective, data-derived benchmark that contrasts with the arbitrary thresholds (30–100 cases), commonly cited in the literature.

The four phases identified demonstrate continuous improvement beyond early competency. Phase I (cases 1–250) represents the initial learning period, characterized by high operative times and variability. Phase II (cases 251–669) marks rapid improvement as techniques become standardized. Phase III (cases 670–1000), following the CUSUM inflection, represents consolidation where above-target performance becomes consistent. Phase IV (cases 1001–1446) achieves mastery-level efficiency with mean operative times of 101.5 min.

The 37.4% overall reduction in operative time (154.6 to 96.8 min) exceeds the 18–25% improvements reported in comparable series. Importantly, statistically significant improvements continued between all consecutive phases (all *p* < 0.001), suggesting that centers accepting intermediate operative times may have opportunities for further optimization. The finding that six surgeons achieved comparable mastery-phase performance suggests these results reflect institutional systems rather than individual exceptional ability. The CUSUM curve presented is an institutional learning trajectory aggregated across six surgeons with heterogeneous baseline experience (range: 0–15 years of laparoscopic surgery at program initiation in 2015). Individual surgeon learning curves were not stratified in this analysis. However, the convergence of all participating surgeons toward comparable mastery-phase operative times (<110 min) suggests that institutional factors—standardized 12-step protocol, dedicated operative team, case volume concentration—may exert greater influence on outcomes than individual baseline proficiency. This interpretation aligns with the volume-outcome literature demonstrating that high-volume institutional systems can mitigate individual surgeon variability [20,21,22,23,24,25]. Future analyses could stratify CUSUM trajectories by surgeon to determine whether baseline laparoscopic experience accelerates individual progression through learning phases.

However, we acknowledge that the extended learning curve (669 cases to inflection, >1000 cases to mastery) may reflect our specific institutional pathway and cannot be directly extrapolated to other settings. Centers with different case volumes, training structures, or technical approaches may demonstrate different trajectories.

**Institutional Volume as a Confounder**

The favorable outcomes observed in this series cannot be attributed solely to surgical technique. Our institution performs approximately 150 donor nephrectomies annually, placing it among the highest-volume centers globally. This volume concentration provides multiple advantages that extend beyond surgical repetition: a dedicated and experienced operative team with consistent personnel, streamlined perioperative protocols refined through thousands of iterations, immediate availability of specialized equipment, and rapid institutional learning from any adverse events. The finding that six different surgeons achieved similar mastery-phase outcomes suggests that institutional systems—rather than individual surgeon expertise—may be the primary driver of performance.

**We caution that these results may not be achievable in lower-volume centers (<50 cases annually) even with the adoption of the identical 12-step technical protocol.** The protocol itself represents only one component of a complex institutional system; its effectiveness likely depends on supporting infrastructure, including experienced anesthesia and nursing teams familiar with the procedure, immediate access to vascular staplers and the GelPOINT^®^ platform, established pathways for graft transfer to recipient teams, and an institutional culture prioritizing donor safety. Centers with lower annual volumes should anticipate longer learning trajectories, potentially different outcome profiles, and may require proportionally more years to achieve comparable case thresholds (e.g., 669 cases to inflection point). Multi-center validation across heterogeneous volume strata is required before these results can be generalized. We therefore present this experience as a benchmark achievable under optimal high-volume conditions rather than as a universally attainable standard.

**This extended learning trajectory appears to translate directly to superior clinical outcomes.** Our operative outcomes compare favorably with major contemporary series. While Takagi’s high-volume analysis reported 195 ± 52 min (*n* = 1895) [3], the Italian multicenter experience 210–240 min with 8–18% complications [4], and Munoz Abraham’s robotic series 208 ± 45 min with 9.05% complications [2], our mean operative time of 105.2 min suggests potential efficiency gains through our approach. Zaytoun reported shorter times (72.8 ± 16.2 min), though with a 2.72% conversion rate [5]. Our comprehensive safety metrics appear encouraging across multiple parameters: mean blood loss of 85.3 mL compared to typically reported ranges of 150–300 mL, absence of intraoperative complications versus published rates of 2–5% for vascular injuries [6,7], and an overall postoperative complication rate of 6.0% with only 0.5% major complications. The mean hospital stay of 3.5 days with early ambulation within 6–8 h demonstrates favorable recovery patterns compared to 4–5 days commonly reported [3,8]. Our warm ischemia time of 3.8 ± 1.2 min aligns with the best reported outcomes, while the absence of hernias at 3-year follow-up contrasts notably with published rates of 1.8–3% [9]. The systematic tracking of orchalgia in male donors (50% at 1 month, declining to 7% at 5 years), also reported in our published paper by Gravetz et al. [26], provides valuable long-term data rarely reported with such precision, suggesting favorable quality of life outcomes [10].

**Recipient Outcomes and Procurement Quality**

The recipient outcomes observed in this series provide indirect validation of procurement technique quality. The DGF rate of 1.0% compares favorably with the 15–25% typically reported in living donor kidney transplantation and likely reflects the consistently short warm ischemia time (3.8 ± 1.2 min) achieved during the mastery phase. Importantly, both DGF cases were attributable to recipient-side factors—severe recipient arterial calcification and technical revision of the recipient anastomosis—rather than graft procurement quality. The low ureteral complication rate in recipients (1.0%) supports the efficacy of our periureteric tissue preservation protocol, which emphasizes maintenance of the “meso-ureter” vascular network and avoidance of direct thermal energy application within 5 mm of the ureteral wall [20,21,22,23]. One-year graft survival of 98.5% and a mean serum creatinine of 1.28 mg/dL align with optimal outcomes reported from high-volume living donor programs. While these recipient data were not the primary focus of this analysis, they suggest that mastery-phase procurement efficiency translates to favorable graft outcomes.

**While these outcomes are encouraging, several important limitations warrant consideration.**

First, this is a single-center retrospective analysis, and outcomes reflect institutional factors developed over 10 years that may be difficult to replicate. The lack of a comparative control group prevents causal inference about the contribution of specific technical elements versus institutional experience.

Second, our 99.5% left kidney procurement rate represents an extreme institutional preference that substantially simplifies the procedure and limits generalizability to right-sided nephrectomy. Centers with different laterality practices may achieve different outcomes.

Third, high institutional volume (approximately 150 cases annually) is a profound confounder. The outcomes reported likely reflect the combined effects of technique, team experience, case selection, and system optimization. These factors cannot be disentangled in this analysis.

Fourth, recipient outcomes (graft function, delayed graft function, and graft survival) represent an important correlate of procurement quality. While our primary focus was donor safety and surgical efficiency, we have provided a summary of first-year recipient outcomes in response to reviewer feedback. However, longer-term recipient follow-up and formal correlation between donor procurement parameters and recipient graft function were beyond the scope of this technical report. The recipient data presented should be interpreted as descriptive rather than as evidence of causal relationships between surgical technique and graft outcomes.

Fifth, this manuscript does not include a video supplement demonstrating the surgical technique. Additionally, our protocol does not include routine baseline scintigraphy, relying on CT angiography for anatomical assessment.

Finally, the learning curve analysis, while using CUSUM methodology, reflects our specific institutional trajectory. The phase boundaries identified (cases 250, 669, and 1000) may not generalize to centers with different volumes, training structures, or technical approaches. Additionally, individual surgeon learning curves were not analyzed. The aggregate institutional CUSUM curve may mask heterogeneity in individual progression rates related to baseline laparoscopic experience, case-mix differences between surgeons, or temporal changes in technique standardization

**Implications for Program Development**

Despite these limitations, our experience offers insights for program development. Our CUSUM analysis suggests that concentrating sufficient annual volume (≥50 cases) may facilitate progression through extended learning curves. The inflection point at case 669 and mastery achievement beyond 1000 cases indicate that proficiency development extends well beyond the 30–100 cases commonly cited. Centers with lower volumes should anticipate longer learning trajectories regardless of the technique adopted.

The ethical dimension remains paramount. Healthy individuals undergo surgery solely to benefit others, warranting the highest standards of care [27]. Viewing any adverse event as an opportunity for systematic improvement, rather than isolated failure, may be essential for achieving consistent outcomes [28,29].

**Future Directions**

Future research should focus on multi-center validation of CUSUM-derived proficiency thresholds, formal cost-effectiveness analysis comparing laparoscopic and robotic approaches, recipient outcome correlation with donor surgical parameters, and long-term donor renal function studies beyond 5 years.

## 5. Conclusions

This single-center analysis of 1446 consecutive laparoscopic living donor nephrectomies demonstrates favorable outcomes within a high-volume institutional setting. CUSUM-based learning curve analysis identified an inflection point at case 669, with mastery-phase operative times of 96.8 ± 25.5 min achieved after approximately 1000 cases. We describe a standardized technique emphasizing the GelPOINT^®^ platform and offset Pfannenstiel incision; however, the outcomes likely reflect the combined effects of institutional volume, team experience, and system optimization rather than technique alone. The high institutional volume (~150 cases annually) represents a significant confounder; centers with lower volumes may not achieve comparable outcomes through technique adoption alone. Multi-center validation is required before generalizing these results.

## Figures and Tables

**Table 1:** Donor demographics and baseline characteristics (*n* = 200).

Variable	Recent 200 Cases (2023–2025)
Age, years	
Mean ± SD	41.6 ± 8.6
Median (IQR)	42 (35–48)
Range	25–66
Sex, *n* (%)	
Male	86 (43.0)
Female	114 (57.0)
BMI, kg/m^2^	
Mean ± SD	24.8 ± 3.0
<25, *n* (%)	102 (53.4)
25–30, *n* (%)	83 (43.5)
>30, *n* (%)	6 (3.1)
Relationship to Recipient, *n* (%)	
Related donor	145 (76.0)
Spouse	35 (18.3)
Altruistic	11 (5.7)
Comorbidities, *n* (%)	
Hypertension	1 (0.5)
Diabetes mellitus	0 (0)
Smoking history	12 (6.3)
Asthma	6 (3.1)
Thyroid disease	4 (2.1)
Previous Abdominal Surgery, *n* (%)	47 (24.6)
Preoperative eGFR, mL/min/1.73 m^2^	
Mean ± SD	95.2 ± 15.8
Preoperative Creatinine, mg/dL	
Mean ± SD	0.85 ± 0.18

**Table 2:** Operative characteristics and outcomes (*n* = 200).

Variable	Recent 200 Cases (2023–2025)
Kidney Side Procured, *n* (%)	
Left	199 (99.5)
Right	1 (0.5)
Renal Vascular Anatomy, *n* (%)	
Single renal artery	164 (86.3)
Multiple renal arteries	26 (13.7)
- 2 arteries	22 (11.6)
- 3 arteries	4 (2.1)
Multiple renal veins	3 (1.6)
Operative Time, min	
Mean ± SD	105.2 ± 28.4
Median (IQR)	100 (85–120)
Range	65–185
Warm Ischemia Time, min	
Mean ± SD	3.8 ± 1.2
Median (IQR)	4 (3–4)
Estimated Blood Loss, mL	
Mean ± SD	85.3 ± 45.2
Median (IQR)	75 (50–100)
Length of Stay, days	
Mean ± SD	3.5 ± 1.8
Median (IQR)	3 (2–4)
Intraoperative Events, *n* (%)	
Conversion to open	0 (0)
Vascular injury	0 (0)
Bowel injury	0 (0)
Spleen injury	0 (0)
Intraoperative bleeding requiring intervention	0 (0)
Extraction Site	
Pfannenstiel (suprapubic)	200 (100)
Surgical Approach	
Complete laparoscopic	200 (100)
Hand-assisted	0 (0)

**Table 3:** Postoperative complications and outcomes (recent 200 cases).

Variable	*n* = 200	Percentage (%)
Overall Complications	12	6.0
Clavien–Dindo Classification		
Grade I	8	4.0
Grade II	3	1.5
Grade ≥ III	1	0.5
Specific Complications		
Wound-Related		
Superficial SSI	2	1.0
Deep SSI	0	0
Seroma	1	0.5
Hernia Complications (3-year follow-up)		
Trocar site hernia	0	0
Pfannenstiel hernia	0	0
Intra-abdominal		
Bleeding requiring intervention	0	0
Bowel obstruction	0	0
Intra-abdominal collection	1	0.5
Urological		
Urinary retention	3	1.5
Urinary tract infection	4	2.0
Pulmonary		
Pneumonia	0	0
Pneumothorax	0	0
Thromboembolic		
Deep vein thrombosis	0	0
Pulmonary embolism	0	0
Pain-Related Complications (Males only, *n* = 86)		
Orchalgia (testicular pain)		
At 1 month	40	46.5
At 1 year	31	36.0
At 5 years (mean ± SD)	6 ± 3	7.0 ± 3.5
Chronic pain (>3 months)	3	3.5
Readmissions		
30-day readmission	2	1.0
90-day readmission	3	1.5
Return to Work, days		
Mean ± SD	18.5 ± 8.2	
Median (IQR)	16 (12–24)	

**Table 4:** Comparison with the literature (selected high-volume series).

Study	Year	*n*	Approach	Operative Time (min)	Conversion Rate (%)	Major Complications (%)	Length of Stay (Days)
Current Study	2025	200	Complete Laparoscopic	96.8 ± 25.5	0	0.5	3.5 ± 1.8
Munoz Abraham et al. [2]	2025	250	Robotic	208 ± 45	1.08	9.05	3.8 ± 1.2
Takagi et al. [3]	2021	1895	Mixed approaches	195 ± 52	2.3	4.7	4.2 ± 2.1
Serrano et al. [17]	2016	4000	Mixed approaches	180 ± 45	1.8	3.9	4.8 ± 2.3
University of Florence [18]	2021	36	Robotic	210 ± 38	0	8.3	4.1 ± 1.5
Kourounis et al. (Cochrane) [19]	2024	Meta-analysis	Robotic vs. Laparoscopic	195–220	1.5–2.8	4.5–8.2	3.5–4.8

**Table 5:** CUSUM-derived learning curve phases.

Phase	Definition	Cases	*n*	Mean ± SD (min)	Median (IQR)	Reduction vs. Previous Phase
I	Initial Learning	1–250	250	154.6 ± 35.9	160.5 (126.8–176.4)	—
II	Rapid Improvement	251–669	419	136.7 ± 32.6	131.0 (111.3–166.1)	11.6% (*p* < 0.001)
III	Consolidation	670–1000	331	118.0 ± 30.1	111.0 (94.2–136.6)	13.6% (*p* < 0.001)
IV	Mastery	1001–1446	446	101.5 ± 26.2	100.2 (84.0–115.2)	14.0% (*p* < 0.001)

CUSUM = cumulative sum analysis. Phases determined by CUSUM inflection point (case 669) and moving average trajectory. IQR = interquartile range. *p*-values from an independent samples *t*-test comparing consecutive phases.

## Data Availability

The data supporting this study are available from the corresponding author upon reasonable request, subject to institutional review board approval and appropriate data sharing agreements.

## Supplementary Materials

The following supporting information can be downloaded at https://www.mdpi.com/article/10.3390/jcm15041363/s1, Figure S1: GelPOINT^®^ Platform Technique: Key Steps in Laparoscopic Living Donor Nephrectomy; Figure S2: “Golden Triangle” (Triangolo d’Oro) dissection and lumbar vein management; Table S1: Recipient outcomes at first post-transplant year (*n* = 200).

## Abbreviations

BMI	Body mass index
GelPOINT	GelPOINT^®^ Advanced Access Platform
HALDN	Hand-assisted laparoscopic donor nephrectomy
IVC	Inferior vena cava
LDN	Laparoscopic donor nephrectomy
