# Supplementary material for: Laparoscopic Living Donor Nephrectomy: Learning Curve Analysis Through 1446 Cases and Outcomes from 200 Consecutive Mastery-Phase Procedures—How I Do It
Source: J Clin Med. 2026 Feb 9;15(4):1363. doi: 10.3390/jcm15041363 (PMC12941911; doi:10.3390/jcm15041363)
Supplement: Supplementary file 1 [file jcm-15-01363-s001.zip › jcm-4115715-supplementary.pdf]

**

Table S1. Recipient Outcomes at First Post-Transplant Year (N = 200)

Variable	N = 200	Percentage (%)
**Graft Function**		
Immediate graft function	198	99.0
Delayed graft function (DGF)*	2	1.0
Primary non-function	0	0
**Graft Survival**		
1-year graft survival	197	98.5
Graft loss (death with functioning graft)	2	1.0
Graft loss (graft failure)	1	0.5
**Renal Function at 1 Year**		
Serum creatinine, mg/dL (mean ± SD)	1.28 ± 0.34	—
eGFR, mL/min/1.73m² (mean ± SD)	58.4 ± 14.2	—
**Surgical Complications**		
Ureteral complications (leak or stricture)	2	1.0
Vascular complications requiring intervention	1	0.5
Lymphocele requiring intervention	3	1.5
Wound complications	4	2.0

*DGF defined as requirement for dialysis within the first week post-transplant. Neither DGF case was attributable to procurement technique (see text).
eGFR = estimated glomerular filtration rate calculated using CKD-EPI equation.

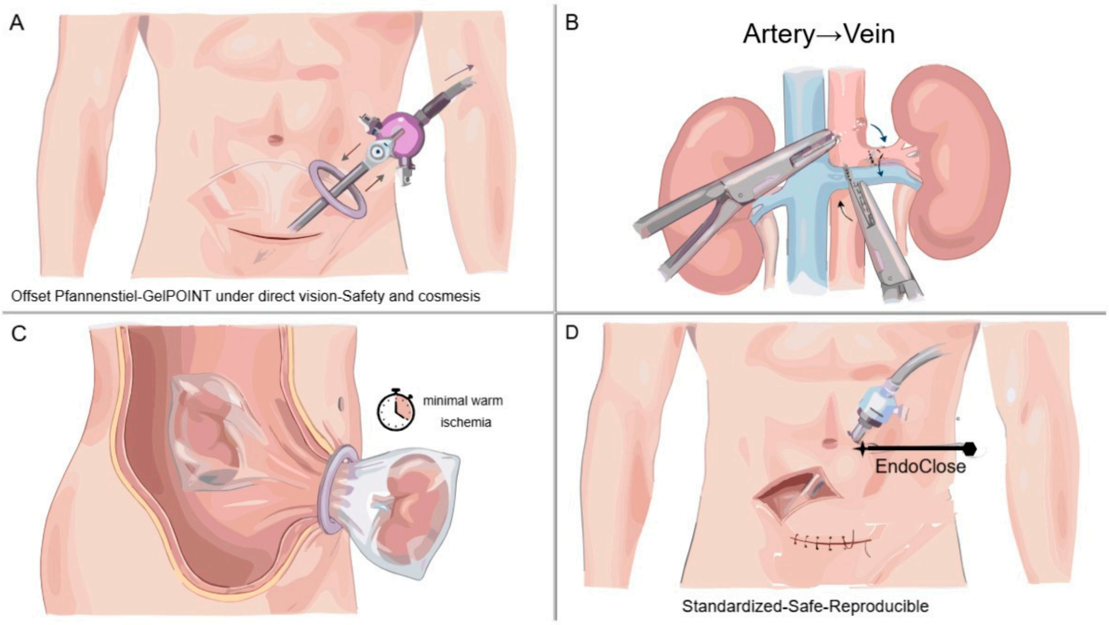

Figure S1. GelPOINT® Platform Technique: Key Steps in Laparoscopic Living Donor Nephrectomy

(A) Offset Pfannenstiel incision with GelPOINT® Advanced Access Platform insertion under direct vision. The skin incision is placed 1 cm cephalad to the pubic symphysis for optimal cosmesis, while the fascial entry is positioned 2–3 cm higher to minimize bladder injury risk. Trocar placement

through the GelSeal® cap provides working ports while maintaining pneumoperitoneum. (B) Hilar vascular control sequence: renal artery stapling precedes renal vein division (Artery→Vein) to minimize warm ischemia. Vascular staplers are positioned with adequate margins from the aortic and caval origins. (C) Kidney extraction through the GelPOINT® platform with minimal warm ischemia. The endobag containing the graft is delivered atraumatically through the suprapubic incision, permitting immediate cold perfusion. (D) Fascial closure under maintained pneumoperitoneum using the EndoClose device, ensuring secure port-site closure and hernia prevention. This standardized, safe, and reproducible technique has been refined over 1,446 consecutive cases.

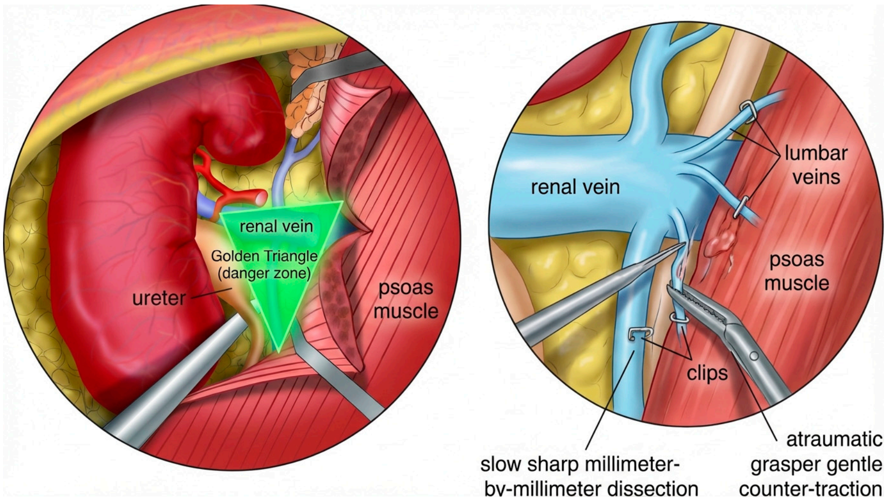

Figure S2. "Golden Triangle" (Triangolo d'Oro) Dissection and Lumbar Vein Management
Left panel: Anatomical boundaries of the "Golden Triangle" danger zone. The green triangle is bordered superiorly by the renal vein, laterally by the ureter (retracted), and medially by the psoas muscle. This zone contains lumbar veins that represent the primary source of intraoperative hemorrhage during laparoscopic donor nephrectomy. Right panel: Safe lumbar vein dissection technique. Slow, sharp millimeter-by-millimeter dissection with atraumatic grasper counter-traction permits identification and clip ligation of lumbar veins prior to division. Meticulous technique within this zone is critical for hemorrhage prevention and donor safety.
